# Hypoxia Differentially Affects Healthy Men and Women During a Daytime Nap With a Dose-Response Relationship: a Randomized, Cross-Over Pilot Study

**DOI:** 10.3389/fphys.2022.899636

**Published:** 2022-05-24

**Authors:** Alain Riveros-Rivera, Thomas Penzel, Hanns-Christian Gunga, Oliver Opatz, Friedemann Paul, Lars Klug, Michael Boschmann, Anja Mähler

**Affiliations:** ^1^ Center for Space Medicine and Extreme Environments Berlin, Institute of Physiology, Charité–Universitätsmedizin Berlin, Corporate Member of Freie Universität Berlin, Humboldt-Universität zu Berlin, Berlin, Germany; ^2^ Department of Physiological Sciences, Faculty of Medicine, Pontificia Universidad Javeriana, Bogotá, Colombia; ^3^ Interdisciplinary Center of Sleep Medicine, Charité–Universitätsmedizin Berlin, Corporate Member of Freie Universität Berlin, Humboldt-Universität zu Berlin, Berlin, Germany; ^4^ Experimental and Clinical Research Center, A Cooperation Between the Max-Delbrück Center for Molecular Medicine in the Helmholtz Association and Charité Universitätsmedizin Berlin, Berlin, Germany; ^5^ Charité–Universitätsmedizin Berlin, Corporate Member of Freie Universität Berlin and Humboldt-Universität zu Berlin, Experimental and Clinical Research Center, Berlin, Germany; ^6^ Max Delbrück Center for Molecular Medicine in the Helmholtz Association (MDC), Berlin, Germany; ^7^ DZHK (German Centre for Cardiovascular Research), Partner Site Berlin, Berlin, Germany

**Keywords:** napping, sleep, hypoxia, high altitude (low air pressure), autonomic nervous system, physiological stress

## Abstract

**Context:** The use of daytime napping as a countermeasure in sleep disturbances has been recommended but its physiological evaluation at high altitude is limited.

**Objective:** To evaluate the neuroendocrine response to hypoxic stress during a daytime nap and its cognitive impact.

**Design, Subject, and Setting:** Randomized, single-blind, three period cross-over pilot study conducted with 15 healthy lowlander subjects (8 women) with a mean (SD) age of 29(6) years (Clinicaltrials identifier: NCT04146857, https://clinicaltrials.gov/ct2/show/NCT04146857?cond=napping&draw=3&rank=12).

**Interventions:** Volunteers underwent a polysomnography, hematological and cognitive evaluation around a 90 min midday nap, being allocated to a randomized sequence of three conditions: normobaric normoxia (NN), normobaric hypoxia at FiO_2_ 14.7% (NH15) and 12.5% (NH13), with a washout period of 1 week between conditions.

**Results:** Primary outcome was the interbeat period measured by the RR interval with electrocardiogram. Compared to normobaric normoxia, RR during napping was shortened by 57 and 206 ms under NH15 and NH13 conditions, respectively (*p* < 0.001). Sympathetic predominance was evident by heart rate variability analysis and increased epinephrine levels. Concomitantly, there were significant changes in endocrine parameters such as erythropoietin (∼6 UI/L) and cortisol (∼100 nmol/L) (NH13 vs. NN, *p* < 0.001). Cognitive evaluation revealed changes in the color-word Stroop test. Additionally, although sleep efficiency was preserved, polysomnography showed lesser deep sleep and REM sleep, and periodic breathing, predominantly in men.

**Conclusion:** Although napping in simulated altitude does not appear to significantly affect cognitive performance, sex-dependent changes in cardiac autonomic modulation and respiratory pattern should be considered before napping is prescribed as a countermeasure.

## 1 Introduction

Sleep is a cornerstone for maintaining physiological homeostasis. Modifications of sleep due to changes in schedule, duration, dark-light cycle, and oxygen availability may result in decreased physical and cognitive performance (Wu et al., 2010; Bloch et al., 2015; Souissi et al., 2020). Among these factors, oxygen availability, particularly in the form of hypobaric hypoxia, calls for more attention given the increasing exposure of humans to such environments. According to the International Air Transport Association, 4,543 million people air-traveled worldwide in 2019 (IATA, 2020), with 12 million people daily exposed to cabin atmosphere corresponding to 2,438 m above sea level, a condition that could significantly decrease oxygen saturation (Humphreys et al., 2005; Muhm et al., 2009). Indeed, it has recently been shown that nighttime sleep in these circumstances induces hypoxia (Elmenhorst et al., 2022). Other causes for the increase in exposure to hypoxic environments come from two distant phenomena, namely global warming, and sociopolitical instability, resulting in migration to high-land cities that may continue in the years to come (Camargo et al., 2020; Hauer et al., 2020). Thus, fully understanding the connection between exposure to hypoxic environments, sleep quality and physical and cognitive performance, are key to developing strategies to face this growing challenge.

Latterly, the impact of daytime napping on sleep health and performance has stood relative to other strategies implemented to complement daily sleep hygiene (Faraut et al., 2017). Daytime napping benefits physiological and mental parameters such as endurance, strength, memory, and attention (Dutheil et al., 2021; Lastella et al., 2021). For instance, healthy adults increased maximal voluntary isometric contraction after 40 or 90 min of a daytime nap vs. no nap, with a concomitant increase in attention evaluated through the digital cancelation test (Boukhris et al., 2020). Also, it has been shown that factual knowledge task is boosted after a 30 min nap with improvement in learning and consolidation processes (Cousins et al., 2019). From the above, an interest arises that associates the implementation of napping as a countermeasure for sleep disturbances characteristic of altitudinal hypoxia. Might daytime napping support cognitive and physical performance during exposure to hypoxic environments?

Over the years, most studies evaluating the effects of hypoxia on sleep have focused on nocturnal sleep (Bloch et al., 2015; Tseng et al., 2015; Horiuchi et al., 2017; Hughes et al., 2017; Steier et al., 2017; Hill et al., 2018; Bird et al., 2021), leaving the impact on daytime sleep under hypoxic environments unclear. Furthermore, direct extrapolations may be inaccurate because of fundamental differences between daytime and nighttime sleep due to circadian changes of essential physiological components such as the endocrine and autonomic systems. For example, the secretion of erythropoietin or cortisol, two key hormones released during hypoxic stress, present higher levels during the day in comparison with the night (Cristancho et al., 2016; Mohd Azmi et al., 2021). Also, the autonomic balance evaluated by LF/HF ratio in a Heart Rate Variability (HRV) analysis has significant differences in a day-night comparison (Boudreau et al., 2012). Nevertheless, important information derived from overnight sleep studies indicates that sex, and divergent lengths and intensity of exposures to hypoxia are critical factors for the degree of hypoxemia reached by the individuals, conditioning physiological and clinical impact (Lombardi et al., 2013). Therefore, prior to its clinical implementation as a countermeasure to nocturnal sleep disturbances, it is necessary to characterize physiologically daytime sleep during hypoxia exposure. Does acute hypoxia alter daytime napping in a dose-and sex-dependent manner? Does acute hypoxia impair cognitive performance after a daytime nap?

Here, our goal was threefold. First, we aimed to characterize the quality and architecture of a daytime nap under two incremental degrees of hypoxia. We monitored napping in a normobaric hypoxia chamber by polysomnographic recording, a standardized and clinically validated methodology, which allows to reliably assess the quality and architecture of sleep (Silber et al., 2007). Although sleep architecture assessment using actimetry or cardiorespiratory monitoring has undergone a remarkable development in recent years (Fekedulegn et al., 2020; Gaiduk et al., 2022), polysomnography remains the gold standard. Despite some limitations, its synchronous and integrated approach of cardiovascular, respiratory, and neural parameters makes it the most objective, sensitive, and specific assessment of sleep duration and its phases (Lee et al., 2022).

Second, we aimed to evaluate the neuroendocrine response to hypoxic stress before, during and after a daytime nap. We implemented HRV analysis using the ECG recordings obtained from the polysomnography (PSG). HRV is a well described proxy of cardiac autonomic balance due to the dynamic and complex interactions between the sympathetic and parasympathetic loops with the sinoatrial node (Heart rate variability, 1996; MacDonald et al., 2020). We further corroborated and correlated the autonomic parameters with blood markers of the hypoxic stress response including catecholamines, cortisol, and erythropoietin (Dimai et al., 2000; Mackenzie et al., 2008). Integrating both neuroendocrine components is ideal for highlighting the coherence between neural and humoral factors triggered during the allostatic response to the hypoxic stress.

Third, we aimed to test the cognitive effect of napping under normobaric hypoxia. We focused on attention, vigilance, and memory since previous studies have shown that these cognitive parameters are affected after sleeping under hypoxic conditions. (De Aquino Lemos et al., 2012). Thus, we conducted psychological evaluations including Digit Span and Stroop Color word tests and the Psychomotor Vigilance Task. To our knowledge, this is the first time that an integrated approach is provided for characterizing daytime napping under normobaric hypoxia conditions.

## 2 Materials and Methods

### 2.1 Study Design

This was a single-blind, randomized, cross-over pilot study conducted at the Experimental and Clinical Research Center of Charité Universitätsmedizin Berlin from October 2019 to December 2020 (ClinicalTrials.gov 31/10/2019, NAPOXIA study, identifier: NCT04146857). Since the main outcomes are more stable within-volunteers than between volunteers, a cross-over design was chosen. Because hypoxia-related symptoms due to three exposures over 3.5 h are mild and usually do not extend beyond 24 h, a significant dropout rate and a long carryover effect were not expected. Consequently, we chose a washout period of 1 week. Randomization of the treatment sequence was done with Randomizer^®^ v. 2.1.0 (Research Randomizer, RRID:SCR_008563), setting sex as factor and minimization as method. Volunteers were blinded to the treatment allocation until the end of the study. They signed informed consent before their participation. The study followed the regulations of the currently applicable version of the Declaration of Helsinki and respective German legislation. The institutional ethics board of Charité Universitätsmedizin Berlin approved the study protocol (EA1/226/19).

### 2.2 Subjects

**FIGURE 1:**
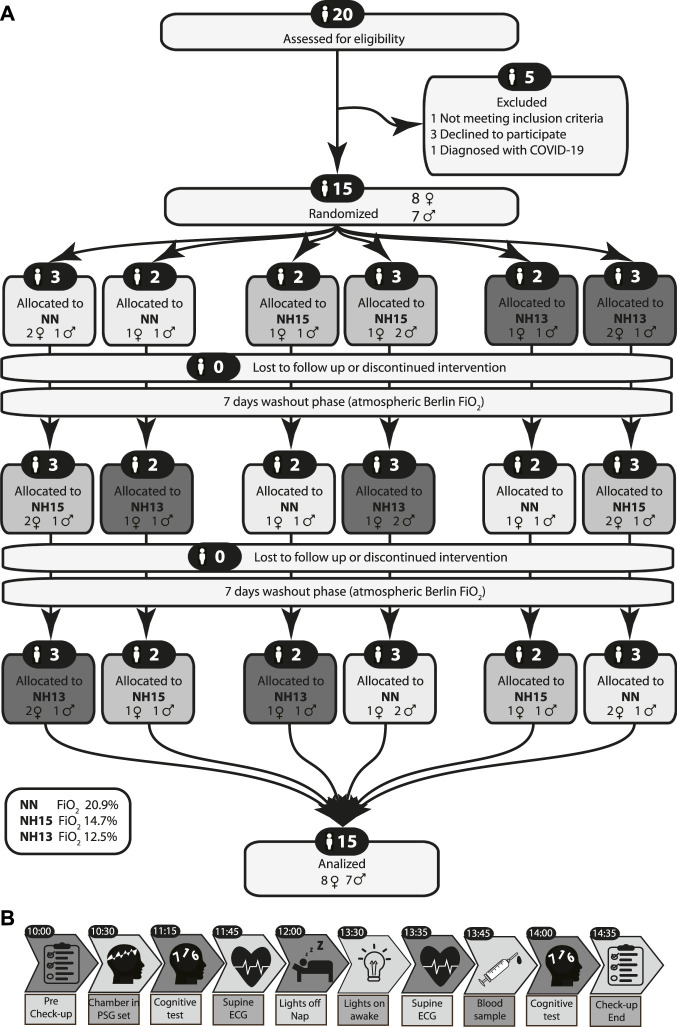
CONSORT 2010 flow diagram **(A)** and study protocol **(B)**.

Healthy volunteers were recruited and screened (Figure 1A) by physical examination, blood tests, bioelectrical impedance analysis (BIA), and electrocardiogram. Key inclusion criteria were: women and men, age 20–45 years and body mass index 20–28 kg/m^2^. Key exclusion criteria were: native highlanders, altitude exposure >2,500 m above sea level (asl) 6 months and plane travel 2 weeks before enrollment, smoking, cardiac or pulmonary diseases, insomnia, and obstructive sleep apnea.

### 2.3 Hypoxia Chamber

Normobaric hypoxia was produced by the nitrogen dilution technique according to methods described previously (Klug et al., 2018; Mähler et al., 2018). In brief, a 38 m^3^ chamber with the Berlin baseline (35 m asl, FiO_2_ = 20.9%) was insufflated with nitrogen gas to decrease the oxygen percentage and simulate 2,660 m asl (FiO_2_ = 14.7%) or 4,000 m asl (FiO_2_ = 12.5%). Environmental parameters such as percentage oxygen and carbon dioxide content, temperature, barometric pressure, and humidity were continuously monitored.

### 2.4 Bioelectrical Impedance Analysis

Body composition was obtained from bioelectrical multifrequency segmental impedance analysis using the BIACorpus RX Spectral (MEDICAL Healthcare GmbH, Karlsruhe, Germany).

### 2.5 Sleep Questionnaires

A validated German version of the Insomnia Severity Index (ISI) was used for insomnia screening (Gerber et al., 2016). Volunteers with clinical insomnia (>15 points) were excluded. Risk of Obstructive Sleep Apnea was evaluated by the German version of the STOP-bang questionnaire downloaded from the official STOP-bang website. Scoring more than 2 points was considered as an exclusion criterion.

### 2.6 Actimetry

We instructed volunteers to follow a 2 h-sleep restriction protocol the night before each experiment, meaning they had to go to bed 2 h later than usual but get up at their usual time. Previous reports using this approach have demonstrated an increase in the sleep drive in a dose-response relationship (Van Dongen et al., 2003). We chose delayed sleep rather than early awakening to ensure volunteers adherence to the protocol. To verify compliance with these instructions and detect sleep modifications around the experiments, volunteers wore an actimeter on their non-dominant wrist for 3 days before and after each experiment (ActiGraph GT3X, Pensacola, FL, United States; ActiGraph Activity Monitor Devices, RRID:SCR_008399). Collected data were processed using ActiLife v. 6.13.3 (ActiGraph, Pensacola, FL, United States) and sleep parameters obtained using the Sadeh algorithm on 60-s epochs.

### 2.7 Polysomnography and Sleep Staging

All volunteers underwent diurnal polysomnographic monitoring (Neurovirtual BWIII PSG Plus Sleep System™, Fort Lauderdale, FL, United States) in accordance with the Manual for the Scoring of Sleep and Associated Events by the American Academy of Sleep Medicine, v. 2.6. Scoring and sleep staging was manually conducted using the BWAnalysis software v 1.98.0.98 (Fort Lauderdale, FL, United States). Electrophysiological signals were sampled at 2,000 Hz, stored at 500 Hz and pass-band filtered at 0.03–35 Hz (electroencephalogram), 0.03–100 Hz (electrocardiogram) and 10–70 Hz (electromyogram).

### 2.8 Electrocardiography and Heart Rate Variability Analysis

Electrocardiographic signals were exported to LabChart Pro software v. 8.1.16 (ADInstruments, Castle Hill, NSW, Australia; Data Acquisition Systems for Life Science, RRID:SCR_001620). Following the HRV Task force guidelines, only sleep stages longer than 5-min were considered for HRV analysis. The HRV Module v. 2.0.3 algorithm automatically detected RR intervals in 5-min Electrocardiography (ECG) segments. Visual inspection and manual correction were applied in accordance with the HRV Guidelines (Malik et al., 1996). The series of RR intervals were exported to Kubios HRV Standard v. 3.4.2 software (University of Eastern Finland, Kuopio, Finland) (Tarvainen et al., 2014), and 4 Hz interpolation rate was applied to obtain HRV linear and non-linear parameters.

### 2.9 Cognitive Tests

Volunteers took a battery of three computer-based cognitive tests before and after the nap using the PEBL v. 2.1 software [Psychology Experiment Building Language (PEBL), RRID:SCR_014794] (Mueller and Piper, 2014). In the first test, a sequence of three digits was presented and the volunteer had to repeat it in the same order (forward digit span test). In a second test, the sequence presented had to be repeated by the volunteer in reverse order (backward digit span test). Both sequences increased the number of digits up to a maximum of nine. In a third test, words naming colors were presented in different colors. The colors of these words may or may not match the name. The volunteer was asked to inhibit the naming of the words and select the color in which they were presented (Color-word Stroop test). In a fourth and final test, a red dot appeared suddenly at irregular intervals in the center of a black screen. The volunteer was asked to be attentive for 5 min to the black screen and activate a key when the red dot appeared with the shortest possible reaction time [5-min version of the Psychomotor Vigilance Task (PVT) (Loh et al., 2004)]. This tests sequence did not change within and between volunteers.

### 2.10 Hormone Analysis

After the nap, 5 ml of EDTA-blood samples were collected from all volunteers for blood count, dopamine, epinephrine, and norepinephrine measurements. Likewise, serum erythropoietin (EPO) and cortisol levels were measured from 5 ml of centrifuged blood. Centrifugation was done after finishing each experiment session and the sample was properly cooled and immediately delivered to the laboratory for analysis. Low levels of dopamine and epinephrine were reported as categorical parameters. For this reason, we handled them as ordinal parameters with the following categories: dopamine low (<60 ng/L), dopamine high (>60 ng/L), epinephrine low (≤30 ng/L), epinephrine high (>30 ng/L).

### 2.11 Experimental Protocol

Volunteers were instructed to abstain from caffeine and alcohol on the preceding day and from vigorous physical activity on the day of the experiment. Before entering the hypoxic chamber, the general condition, vital signs, quality of sleep, and compliance with the 2-h sleep restriction were evaluated through a general check-up (Figure 1B). Inside the chamber, polysomnography electrodes were positioned. Then sitting on the bed, the volunteers took the pre-sleep cognitive tests. Afterward, a 10-min baseline ECG was recorded while volunteers lay supine and awake. After this, the lights were switched off and volunteers were asked to sleep for 90 min. After a 5-min awakening period, another 10-min ECG was recorded, followed by blood sample collection. Finally, volunteers repeated the cognitive tests. All volunteers repeated this experiment at three conditions in a randomized sequence: normobaric normoxia at FiO_2_ 20.9% (NN), normobaric hypoxia at FiO_2_ 14.7% (NH15), and normobaric hypoxia at FiO_2_ 12.5% (NH13) (Figure 1A).

### 2.12 Outcome Measures

#### 2.12.1 Primary Outcome Measure

Primary outcome measure was the change in the cardiac autonomic balance during or after napping, assessed by RR intervals as a component of HRV under hypoxia vs. normoxia.

#### 2.12.2 Secondary Outcome Measures: Polysomnography

1) Parasympathetic Nervous System Index (PNS) resulting from the computation of mean RR interval, Root mean square of successive RR interval differences (RMSSD) and Poincaré plot index of the Standard Deviation of the instantaneous beat-to-beat variability (SD1) in normalized units
2) Sympathetic Nervous System Index (SNS) resulting from the computation of mean Heart Rate (HR), Baevsky’s stress index (SI) and Poincaré index of the Standard Deviation of the continuous long-term variability (SD2) in normalized units
3) Peripheral Oxygen Saturation (SpO_2_).
4) Sleep Efficiency (SE).
5) Wake After Sleep Onset (WASO).
6) Sleep Onset Latency (SOL).
7) Apnea-Hypopnea Index (AHI).
8) Periodic Breathing Index (PBI).
9) Percentage of REM sleep and stage 1 (N1), stage 2 (N2) and stage 3 (N3) of non-REM sleep.

#### 2.12.3 Secondary Outcome Measures: Hormone Levels

1) Erythropoietin (EPO).
2) Cortisol
3) Norepinephrine
4) Epinephrine
5) Dopamine.

#### 2.12.4 Secondary Outcome Measures: Cognition

1) Length of digit span tests
2) Lapses and reaction time in PVT
3) Accuracy and reaction time in color-word Stroop test

### 2.13 Data Management, Analysis, and Statistical Analysis

Study data were pseudonymized, collected, and managed using REDCap (REDCap, RRID:SCR_003445) electronic data capture tools (Harris et al., 2009) hosted at Charité Universitätsmedizin Berlin. The sample size was determined following the rule of thumb for pilot studies proposed by Julious SA (Julious, 2005). Data graphing were carried out by Origin Pro v. 9.3.226 software (OriginLab Corporation, Northampton, MA, United States; RRID:SCR_014212). Rstudio v. 1.4.1103 (RStudio, RRID:SCR_000432) based on R v. 4.0.4 software (R Project for Statistical Computing, RRID:SCR_001905) and Jamovi v. 1.6.16 (The jamovi project 2021; RRID:SCR_016142) were used for statistical analysis. R packages included ggplot, corrplot and MASS. According to Rubin’s classification, missingness was considered MAR (PSG data) or MACR (blood analysis). Normal distribution was evaluated by Shapiro-Wilk test and QQ plots. Robust statistical processing using Huber M-estimate with winsorized at 1.5 standard deviations and converge tolerance 1e-06 was applied to detect outliers. Descriptive statistics are presented by mean ± SD unless stated otherwise. Ordinal parameters are presented as percentage. Since this was a pilot study, inferential statistics should be understood as an exploratory approach to the data analysis. Multiple comparison test with Holm’s correction is presented only to describe the main patterns, but it is limited for the reasons previously stated. Since within-subject correlations arose from the cross-over design, a linear mixed model approach was chosen. To estimate differences between normoxia and hypoxia, modeling was applied using FiO_2_, sex, or sleep phase as fixed factors and intercept-volunteer as random factor. Normal distribution of residuals was verified. Sex differences in demographic and hematologic parameters at baseline were analyzed by a *t*-test. McNemar’s test of paired contingency tables was performed to analyze epinephrine and dopamine. Correlations between parameters were calculated by Pearson’s r. The overall significance level was set at two-tailed *p* = 0.05.

## 3 Results

### 3.1 Demographic, Haematological, and Environmental Evaluation

Total recruitment, screening, attrition, and allocation are presented in Figure 1. Baseline anthropometric and hematologic characteristics of 15 men and women who completed the study are shown in Table 1. Men were slightly, but not significantly, older than women.

**TABLE 1:** Anthropometric and hematologic parameters of 15 healthy men and women. Data as mean ± SD.

	Women (8)	Men (7)	All (15)	Sex difference p-value (*t*-test)
Age (years)	26.5 ± 3.2	32.6 ± 8.1	29.3 ± 6.6	0.07
Mass (kg)	61.1 ± 4.7	81.9 ± 8.4	70.8 ± 12.5	<0.001
Height (cm)	167 ± 5.0	182 ± 9.8	174 ± 10.8	0.002
Body Mass Index (kg/m^2^)	22.0 ± 1.7	24.8 ± 2.8	23.3 ± 2.6	0.034
Fat Mass (%)	28.3 ± 4.3	22.1 ± 5.0	25.4 ± 5.5	0.023
Fat Free Mass (%)	71.7 ± 4.3	77.9 ± 5.0	74.6 ± 5.5	0.023
Body Water%	50.4 ± 3.5	57.1 ± 4.0	53.5 ± 5.0	0.004
Erythrocytes/pL	4.31 ± 0.2	5.13 ± 0.4	4.69 ± 0.5	<0.001
Hemoglobin (g/dl)	12.8 ± 0.7	15.2 ± 0.8	13.9 ± 1.4	<0.001
Hematocrit (%)	38.5 ± 1.9	45.0 ± 2.8	41.5 ± 4.0	<0.001

In line with our instructions, actimetry showed a 2-h sleep restriction the night before tests. Along with this, there were no changes in sleep efficiency (SE, 87 ± 2%) and wake after sleep onset (WASO, 59 ± 8 min) in the nights before vs. after experiments. This apparent undisturbed sleep could be because the dose of sleep restriction was small and the exposure to hypoxia was short. In addition, similar protocols (such as the splitting sleep protocol) have even found positive effects from their implementation (Cousins et al., 2021).

Environmental parameters within the chamber did not show differences throughout the study (barometric pressure: 759 ± 7 mmHg; humidity: 37.6 ± 6%; temperature: 20.8 ± 1°C).

### 3.2 Primary Outcome Measure

Since REM and N3 were less than 5 min long or absent in more than 30% of experiments, these phases were not included in the HRV analysis. The RR interval before, during, and after nap was shorter under hypoxia vs. normoxia (Table 2). Regarding the analysis by sex, men showed a longer RR interval in all conditions and phases compared to women, being pronounced for N2 in NH15 (*p* = 0.05) but not in NN (*p* = 0.32). This sex difference was reciprocally evident for HR in NH13 N2 (*p* = 0.01), NH13 pos (*p* = 0.02) and NH15 pos (*p* = 0.04) (Table 2). In comparison with N2, a significant shortening in RR was observed after napping in all conditions (*p* < 0.001). A comparison between pre- and post-nap RR interval revealed difference in NH15 (*p* = 0.006) and NH13 (*p* = 0.007), but not in NN (*p* = 0.07).

**TABLE 2:** Heart rate variability parameters during a daytime nap in normobaric normoxia (NN) and normobaric hypoxia (NH15: FiO_2_ 14.7, NH13: FiO_2_ 12.5).

		Phase	N	RR (ms)	RMSSD (ms)	SD1 (ms)	PNS	HR (b.p.m.)	SD2 (ms)	SI	SNS
NN	Women	Pre	8	985 ± 115	66.3 ± 48	47 ± 34	1 ± 1.84	61.6 ± 7.1	64.3 ± 25.7	8.2 ± 2.7	−0.52 ± 0.92
		N2	8	1,024 ± 146	74.9 ± 43	53 ± 30	1.44 ± 1.79	59.8 ± 9.4	64.4 ± 14.8	7.8 ± 3.3	−0.73 ± 1.16
		Pos	8	938 ± 125	56.7 ± 46	40 ± 33	0.55 ± 1.81	65 ± 8.9	50.2 ± 22.1	10.8 ± 4	0.08 ± 1.21
	Men	Pre	7	1,172 ± 170	55 ± 34	39 ± 24	1.54 ± 1.76	52.1 ± 6.9	52.6 ± 15.7	8.7 ± 3.9	−1.05 ± 1.13
		N2	7	1,230 ± 157	48.7 ± 24	35 ± 17	1.64 ± 1.32	49.4 ± 5.5	49.1 ± 19.6	8.5 ± 2.7	−1.26 ± 0.75
		Pos	7	1,083 ± 95	42.8 ± 21	30 ± 15	0.77 ± 0.97	55.8 ± 5	54 ± 23.3	9.5 ± 3.7	−0.65 ± 0.74
	All	Pre	15	1,072 ± 168	61 ± 41.2	43.2 ± 29.2	1.25 ± 1.76	57.2 ± 8.4	58.8 ± 21.8	8.4 ± 3.2	−0.77 ± 1.02
		N2	15	1,120 ± 180	62.7 ± 36.6	44.4 ± 26	1.53 ± 1.54	54.9 ± 9.3	57.3 ± 18.4	8.1 ± 2.9	−0.98 ± 1
		Pos	15	1,006 ± 132	50.2 ± 36.2	35.6 ± 25.6	0.65 ± 1.43	60.7 ± 8.5	52 ± 21.9	10.2 ± 3.8	−0.26 ± 1.05
NH15	Women	Pre	8	876 ± 122	42.1 ± 21	30 ± 15	0.25 ± 1.75	69.6 ± 9.1*	61.8 ± 28.9	9 ± 3.4	0.15 ± 1.02
		N2	7^m^	940 ± 165	64.7 ± 43	46 ± 30	0.75 ± 1.91	65.6 ± 11.4	62.3 ± 23.4	9.4 ± 4.5	−0.07 ± 1.5
		Pos	7^m^	795 ± 109*	26.6 ± 17	19 ± 12	−1 ± 0.94*	76.6 ± 9.6**	37.8 ± 18.9	15.2 ± 6.5	1.61 ± 1.6*^A^
	Men	Pre	6^m^	1,135 ± 185	53.8 ± 37	38 ± 26	1.28 ± 1.88	53.9 ± 8.2	63.6 ± 19.2	7.7 ± 2.5	−1.02 ± 0.94
		N2	6^m^	1207 ± 123	51.7 ± 28	37 ± 20	1.54 ± 1.37	50.1 ± 4.7	67.9 ± 26.9	7.6 ± 2.7	−1.28 ± 0.68
		Pos	6^m^	1,036 ± 86	38.3 ± 25	27 ± 18	0.4 ± 1.03	58.3 ± 5.3	55.7 ± 26.6	10.4 ± 4.7	−0.31 ± 0.85
	All	Pre	13	987 ± 197*	47.5 ± 28.9	33.7 ± 20.5	0.69 ± 1.81	62.9 ± 11.6*	62.6 ± 24.3	8.4 ± 3	−0.36 ± 1.13
		N2	13	1,063 ± 198	58.7 ± 35.7	41.6 ± 25.3	1.12 ± 1.67	58.4 ± 11.8	64.9 ± 24.2	8.6 ± 3.7	−0.63 ± 1.31
		Pos	13	906 ± 157**^A^	32 ± 20.7	22.7 ± 14.6	−0.36 ± 1.19*^A^	68.2 ± 12.2*^A^	46.1 ± 23.7	13 ± 6	0.73 ± 1.6^A^
NH13	Women	Pre	8	781 ± 85**	30.5 ± 17	22 ± 12*	−0.96 ± 0.81**	77.5 ± 7.9**	43.9 ± 15.8	13.4 ± 4.5	1.4 ± 1.14**
		N2	8	797 ± 168**	36.8 ± 32	26 ± 23*	−0.73 ± 1.64**	78.2 ± 16**	48.4 ± 31.4	16.6 ± 12.5*	1.97 ± 2.96**
		Pos	8	708 ± 93**	15.7 ± 11	11 ± 8*	−1.77 ± 0.77**	86 ± 10.7**^A^	31.3 ± 17.5	19.3 ± 7.1*	2.95 ± 1.61**^A^
	Men	Pre	7	1,010 ± 126**	46.8 ± 28*	33 ± 20	0.45 ± 1.29	60.2 ± 7.1	81 ± 41.8	7.7 ± 2.6	−0.55 ± 0.82
		N2	7	1,048 ± 103**	52.3 ± 32*	37 ± 22	0.77 ± 1.31	57.7 ± 5.2*	93 ± 45.6*	7 ± 3.5	−0.81 ± 0.78
		Pos	7	909 ± 50**	27.3 ± 14*	19 ± 10	−0.55 ± 0.59	66.2 ± 3.7*	54.3 ± 18.8	10.8 ± 4	0.34 ± 0.74
	All	Pre	15	888 ± 156**	38.1 ± 23.3*	27 ± 16.5*	−0.3 ± 1.26**	69.4 ± 11.6**	61.2 ± 35.2	10.8 ± 4.7	0.49 ± 1.39*
		N2	15	914 ± 188**	44 ± 31.7	31.2 ± 22.5	−0.03 ± 1.64**	68.6 ± 15.9**	69.2 ± 43.8	12.1 ± 10.4	0.67 ± 2.59**
		Pos	15	802 ± 127**^A^	21.1 ± 13.5*	15 ± 9.6*	−1.2 ± 0.92*^A^	76.7 ± 12.9**^B^	42 ± 21.1	15.4 ± 7.2*	1.73 ± 1.83**^A^

^m^Missing data due to lack of sleep or ECG artifacts. Data as mean ± SD. *p <0.05, **p <0.001 comparing the same phase with NN. ^A^ *p* <0.05, ^B^ *p* <0.001 comparing with pre in the same condition (Linear Mixed Model).

### 3.3 Effects of Hypoxic Stress on Cardiac Autonomic Balance

**FIGURE 2:**
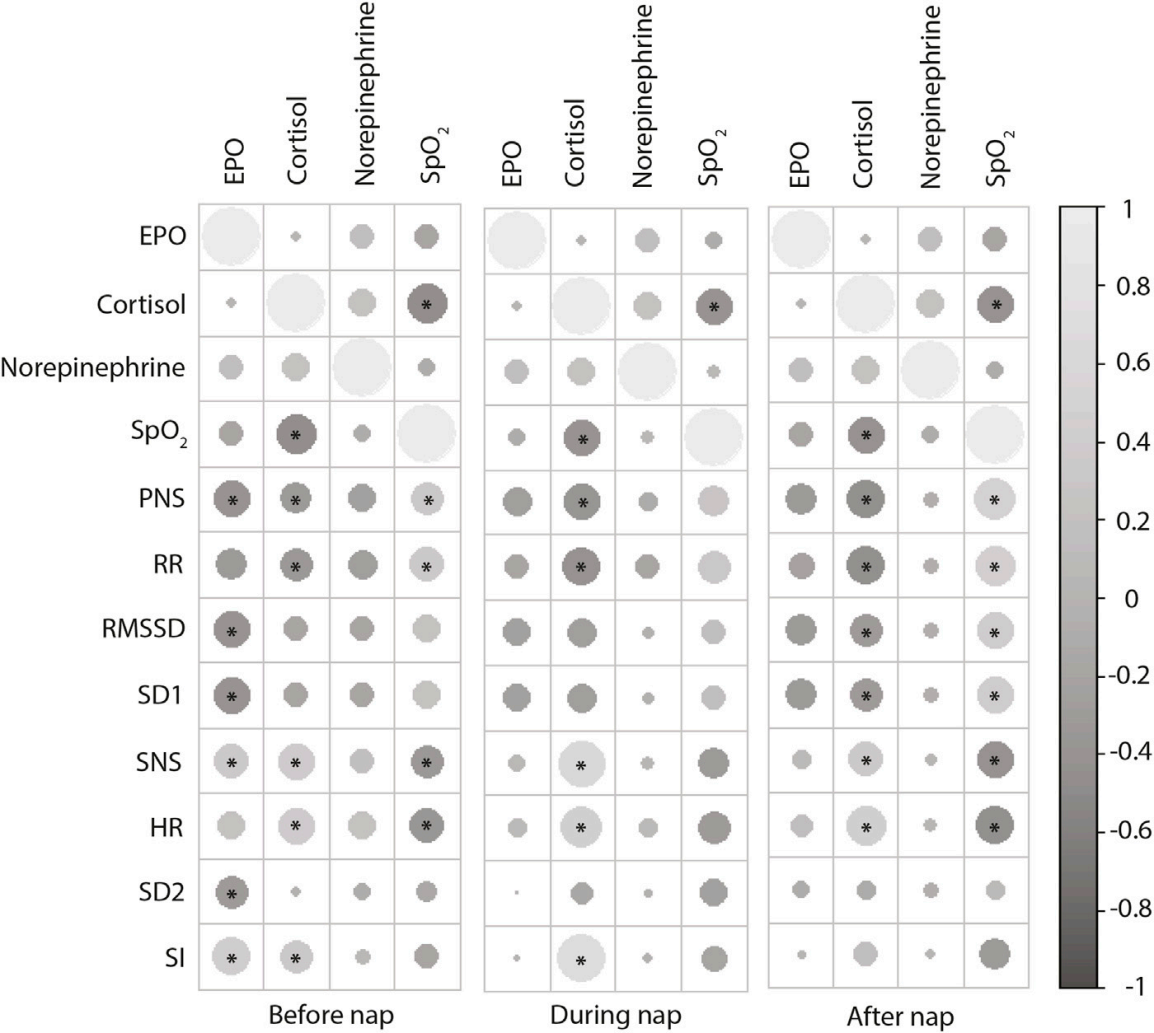
Correlations of after nap stress hormone levels (EPO, cortisol, norepinephrine) with HRV parameters measured after, during and after the nap. Positive correlations are displayed in light gray and negative correlations in dark gray. Circle size and intensity are proportional to the correlation coefficients. *p < 0.05 (Pearson’s r).

The SNS index changed in NH13 vs. NN, particularly in women (Table 2). SNS index was different in women during nap vs. pre-nap, both at NH13 (*p* = 0.019) and NH15 (*p* = 0.03). PNS index showed a similar pattern, but only at NH13 (*p* = 0.04). Finally, peripheral oxygen saturation (SpO_2_) correlated positively with PNS index and RR, both before and after the nap (Figure 2). In line with this, SpO_2_ was negatively correlated with SNS index and HR. Mean and SD of further factors determining PNS index (RMSSD and SD1) and SNS index (HR, SI and SD2) are shown in Table 2.

### 3.4 Effects of Hypoxic Stress on Sleep

A summary of polysomnography (PSG) data is presented in Table 3. Total sleep time (TST), sleep period time (SPT), sleep onset latency (SOL), WASO, and SE were not affected by both hypoxic conditions and sex. Interestingly, N2 percentage of TST increased in NH15 and NH13 vs. NN (*p* < 0.001). Accordingly, N3 percentage of TST decreased in NH15 and NH13 (*p* < 0.001), both vs. NN. All men showed REM sleep in NN (17 ± 4% of TST) but only 4 of 8 women. Those percentages fell in NH13, where women did not get any REM sleep, and only 3 of 7 men did.

**TABLE 3:** Main polysomnography parameters during a daytime nap in normobaric normoxia (NN) and normobaric hypoxia (NH15: FiO_2_ 14.7, NH13: FiO_2_ 12.5).

		N	TST (min)	WASO (min)	SPT (min)	SOL (min)	N1 %TST	N2 %TST	N3 %TST	SE TST	SE SPT	% SpO_2_ TST
NH	Women	8	68.3 ± 21	21 ± 24.6	87.4 ± 7.4	3.9 ± 5.8	24 ± 22.1	39.8 ± 11.5	30.6 ± 20.4	74.7 ± 25.7	94 ± 7.6	96.1 ± 1.6
	Men	7	71.5 ± 18.3	15.6 ± 11.7	84.3 ± 14.2	3.6 ± 3.2	16.7 ± 7.1	42.3 ± 14.8	37.6 ± 21.3	78.9 ± 15.5	93.3 ± 7.5	95.9 ± 1.1
	All	15	69.8 ± 19.2	18.5 ± 19.2	85.9 ± 10.8	3.8 ± 4.6	20.6 ± 16.7	40.9 ± 12.7	33.9 ± 20.4	76.6 ± 20.9	93.6 ± 7.3	96 ± 1.3
NH15	Women	7^m^	83.6 ± 4.9	6.1 ± 3.2	89.4 ± 3.9	2.4 ± 1.9	13.1 ± 4.9	57.2 ± 11.2	18.2 ± 12.6	90.8 ± 4.3	97 ± 2.2	87.7 ± 2.5^**^
	Men	6^m^	60.8 ± 17.4	18.6 ± 15.9	82.5 ± 3.9	4.1 ± 1.8	26.9 ± 12.2	63.4 ± 11.1	9.7 ± 10.6^*^	67.1 ± 19.2	91 ± 4.7	85.8 ± 2.9^**^
	All	13	73.1 ± 16.7	11.9 ± 12.4	86.2 ± 5.2	3.2 ± 2	19.4 ± 11.2	60 ± 11.1^**^	14.3 ± 12.1^**^	79.8 ± 17.7	94.2 ± 4.6	86.8 ± 2.7^**^
NH13	Women	8	62.6 ± 24.4	21.2 ± 20.7	79.1 ± 12.8	5 ± 7.2	20.7 ± 13.5	63.2 ± 13.2^**^	11.6 ± 15.8^*^	70.2 ± 26.5	88.8 ± 12.6	78.4 ± 3.8^**^
	Men	7	62.6 ± 18.7	23.9 ± 16.5	77.6 ± 22.9	5 ± 4.3	23.2 ± 18.6	53.4 ± 16.3	18.7 ± 18.1	69.9 ± 18.3	85.3 ± 17.2	73.1 ± 5.8^**A^
	All	15	62.6 ± 21.1	22.4 ± 18.3	78.4 ± 17.5	5 ± 5.8	21.9 ± 15.5	58.6 ± 15^**^	14.9 ± 16.7^**^	70 ± 22.2	87.1 ± 14.5	75.9 ± 5.4^**^

^m^Missing data due to lack of sleep. TST, Total Sleep Time; WASO, Wake after Sleep Onset; SPT, Sleep Period Time; SOL, Sleep Onset Latency; SE, Sleep Efficiency; SpO_2_, Peripheral oxygen saturation. Data as mean ± SD. *p < 0.05, **p < 0.001 comparing with NN. ^A^ *p* = 0.012 comparing with women in the same condition (Linear Mixed Model).

**FIGURE 3:**
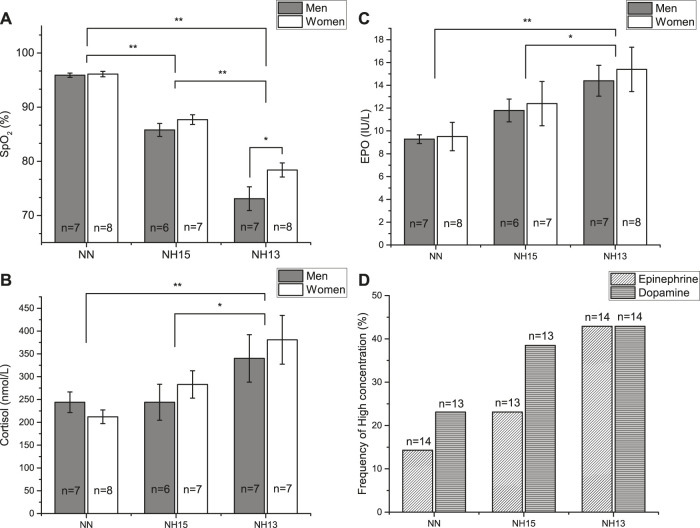
Peripheral oxygen saturation (SpO_2_) **(A)** and cortisol **(B)** and erythropoietin (EPO) **(C)** concentrations after a 90 min nap under normoxic (NN) and hypoxic conditions (NH15: FiO_2_ 14.7, NH13: FiO_2_ 12.5) in healthy men (*n* = 7) and women (*n* = 8). Data as mean ± SEM. *p < 0.05, **p < 0.001 (Linear Mixed Model). Frequency of high concentration of epinephrine and dopamine **(D)**. Missing data due to blood specimen damage during processing.

During the nap, SpO_2_ was different between all three conditions (*p* < 0.001) (Figure 3A). At NH13, SpO_2_ values were higher in women vs. men (*p* = 0.012), but not at NH15 (*p* = 0.629) or NN (*p* = 0.87) (Table 3 and Figure 3A).

**FIGURE 4:**
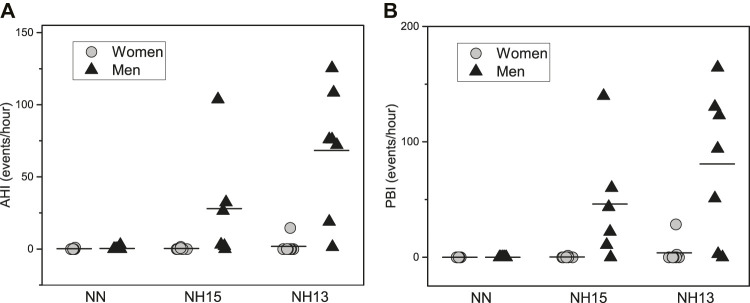
Apnea-Hypopnea Index (AHI) **(A)** and Periodic Breathing Index (PBI) **(B)** during a 90 min nap under normoxic (NN) and hypoxic conditions (NH15: FiO_2_ 14.7, NH13: FiO_2_ 12.5) in healthy men (*n* = 7) and women (*n* = 8).

Finally, the incidence of respiratory events under hypoxic conditions was higher in men vs. women, including central sleep apneas and periodic breathing (Figure 4). Indeed, only one woman presented a periodic breathing pattern in NH13. The reticulocyte production index in NH13 correlated strongly with the number of apneas [r (14) = 0.57, *p* = 0.03] and PB [r (14) = 0.66, *p* = 0.008].

### 3.5 Effects of Hypoxic Stress on Endocrine Response

Erythropoietin (EPO) concentrations raised with increasing hypoxia with no sex-specific differences (Figure 2B). This EPO increase was accompanied by an increment in erythrocytes in NH13 (4.67 ± 0.13/pL; *p* = 0.008), but not in NH15 (4.61 ± 0.11/pL, *p* = 0.074), both vs. NN (4.56 ± 0.12/pL). EPO showed positive correlations with SI and SNS index and negative correlations with RMSSD, SD1, and PNS index, particularly before the nap (Figure 2).

Cortisol concentrations also increased with increasing hypoxia (Figure 3C). In contrast to EPO, cortisol was correlated with HRV parameters during nap, particularly with SI and SpO_2_ (Figure 2).

We found an increased frequency of high concentrations of epinephrine (NN = 14.3%, NH15 = 23.1%, NN13 = 42.9%) and dopamine (NN = 23.1%, NH15 = 38.5%, NH13 = 42.9%), which were, however, not significant (Figure 3D). Norepinephrine concentrations did not change (data not shown).

### 3.6 Effects of Hypoxic Stress on Cognition

Performance in the Forward and Backward Digit Span test before the nap did not change due to hypoxia (Forward, NN: 7.3 ± 1.6, NH15: 6.7 ± 1.6, NH13 = 7.1 ± 2; Backward, NN = 7.4 ± 1.5, NH15 = 6.7 ± 1.1, NH13 = 7.5 ± 1.5). However, there was a strong correlation between cortisol concentrations and the Forward Digit Span test after the nap [r (14) = −0.61, *p* = 0.02] at NH13, but not NH15 or NN.

Incongruent-Congruent response times (I-C) in the Stroop test did not change markedly. Strikingly, I-C were higher under hypoxia (NN: 75.9 ± 85.5 ms, NH15: 128 ± 71.5 ms, NH13: 113 ± 93.6 ms). At NH13, congruence errors were correlated with HRV, including SNS index [r (14) = 0.54, *p* = 0.04], HR [r (14) = 0.53, *p* = 0.04] and SI [r (14) = 0.52, *p* = 0.04]. Response time to the congruent stimulus improved after nap at NN (pre: 737 ± 129 ms vs. pos: 654 ± 118 ms, *p* = 0.035). Importantly, this improvement was not observed under both hypoxic conditions.

Lapses in PVT did not change (NN: 4.1 ± 5.6, NH15: 3.6 ± 5.3, NH13: 3.9 ± 4.3). At NH13, the PVT response time was correlated with EPO [r (14) = −0.59, *p* = 0.02], SpO_2_ during the nap [r (14) = −0.52, *p* = 0.05] and SI after the nap [r (14) = 0.59, *p* = 0.03].

## 4 Discussion

Napping is recommended as an energetic and cognitive booster (Milner and Cote, 2009; McDevitt et al., 2018). Here, we describe the main physiological changes during a daytime nap under simulated altitude conditions, including cardiac autonomic balance, stress hormone secretion, and respiratory pattern. We assessed whether the presented neuroendocrine and oxygenation changes affect cognitive performance in attention, memory, and vigilance functions. We investigated 15 healthy adult volunteers who took a short daytime nap under two hypoxic conditions.

Primary outcome measure was the change in the RR Interval under hypoxic conditions. This component of the electrocardiogram is directly correlated with cardiovagal activity (Chapleau and Sabharwal, 2011). Under normoxia we found values similar to the general population (926 ± 90 ms, resting, normoxic) (Nunan et al., 2010). Sleep was accompanied by an RR elongation that has been reported both in nighttime (Herzig et al., 2018) and daytime sleep (Cellini et al., 2018). The evident decrease in SpO_2_ under both hypoxic conditions was accompanied by a RR shortening, which affected both awake and sleep measurements. This effect aims to improve oxygen delivery and is mediated by the carotid bodies in hypoxic conditions and could be explained by a depression of cardiovagal activity *via* nucleus ambiguous or a sympathetic stimulation via the rostral ventrolateral medulla (Zera et al., 2019). During hypoxic stress, the RR interval after the nap was shorter than before, showing inertia after awakening in the increased sympathetic activity. This contrasts with the normoxic condition in which the values did not change. Although the RR interval was consistently longer in men than in women, this difference was accentuated during sleep under hypoxia. This sex-dependent difference could be due to discrepancies in breathing control, particularly in chemosensitivity to hypoxia (Gargaglioni et al., 2019; Littlejohn et al., 2020).

The cardiac autonomic change evident by RR shortening was consistent with changes of cardiovagal markers in HRV, such as the RMSSD, SD1, and the PNS index. These findings coincide with Boos et al. (Boos et al., 2017), who also found changes in parasympathetic markers dependent on sex and altitude in awake subjects early at the morning. Evaluation in field conditions after acute exposure to hypoxia at 3,150 m also showed similar changes before and after sleep (Yih et al., 2017). This decrease in cardiovagal tone could be accompanied by either a relative (i.e., no actual increase in activity) or an absolute increase in sympathetic activity. Since the increments in HR, SI, and SNS index were accompanied by an increase in the frequency of high levels of epinephrine during hypoxia, the results of this study rather point to the second possibility. Contrary to Panjwani et al. (2006), who found an increase in plasma norepinephrine after a simulated ascent to 3,500 m, our study did not show such changes. The explanation for this dissimilarity of responses could be that in our study subjects were evaluated at rest or that noradrenergic mediation is more neural than endocrine, as Rowel et al. (1989) have shown. It has also been found that although an increased sympathetic influx accompanies acute hypoxia, norepinephrine clearance is equally increased (Leuenberger et al., 1991).

In any case, this absolute or relative increase in sympathetic activity was more pronounced in women than in men, with the aforementioned post-nap inertia. This is paradoxical considering that men exhibited the lowest SpO_2_ values. However, there is evidence that women have a different sensitivity to hypoxia (Gargaglioni et al., 2019), probably due to the effects of female hormones on the neural circuits controlling cardio-ventilatory responses (Littlejohn et al., 2020). Additionally, men presented periodic breathing, a phenomenon that has been linked to a RR lengthening as a consequence of increment in tidal volume and its effect on vagal tone (Insalaco et al., 2016).

Periodic breathing due to altitudinal hypoxia is a respiratory event that has been previously described both under field (Ju et al., 2021) and laboratory conditions (Pramsohler et al., 2019) during nighttime sleep. Here, we confirm this during a daytime nap and a short hypoxia exposition. As during its nocturnal presentation, periodic breathing has an accentuated sex-dependent incidence, being predominantly a male phenomenon. This could be explained either by an inhibitory effect of female hormones or a trigger effect of testosterone. Although the two possibilities are not mutually exclusive, some results of this study favor the second possibility. On the one hand, the only woman who presented periodic breathing has been diagnosed with polycystic ovary syndrome (POS), a condition in which testosterone levels are increased substantially (Lerchbaum et al., 2014). Likewise, the man who had fewer episodes of periodic breathing was the oldest one and, therefore, might have lower testosterone levels (Ferrini and Barrett-Connor, 1998). Studies evaluating nocturnal sleep have shown that although in a lower proportion than men, women also develop periodic breathing, especially at higher altitudes (Lombardi et al., 2013; Caravita et al., 2015). A notable difference in our daytime sleep findings is that no women presented periodic breathing (excluding the one with POS). These differences could be either due to infradian phenomena (the phase of the menstrual cycle in which women are evaluated), circadian phenomena (the contrast between daytime and nighttime sleep) or to a time-dependent mechanism in which only prolonged exposure triggers periodic breathing in women. Studies discriminating whether one or all these mechanisms are involved are needed.

Although there are differences between nighttime and daytime sleep, in normoxic experiments a similar distribution of N2 has been described previously (Jiang et al., 2018). Our study corroborates that, like nighttime sleep, a daytime nap in normoxia is accompanied by a higher proportion of N2 sleep followed by N3 and REM sleep. Similar to men evaluated during nighttime sleep in a simulated altitude of 2,000 m (Hoshikawa et al., 2007), we found an increased proportion of N2 and decreased proportions of N3 and REM sleep under hypoxic conditions. Considering that cerebral clearance has been associated with sleep (Xie et al., 2013) and particularly its slow waves (Fultz et al., 2019), a decrease in the N3 proportion may affect brain function. Indeed, this has been pointed out as the link between high-altitude hypoxia and cerebral edema (Simka et al., 2018). Besides hypoxia, we cannot rule out a small additive effect of factors such as sleep deprivation or caffeine withdrawal (Wu et al., 2010; Weibel et al., 2020).

As previously described (Coste et al., 2005; Keenan et al., 2020), the endocrine response to hypoxic stress was led by cortisol, which showed a positive correlation with sympathetic HRV markers, particularly during the nap. Indeed, a mutually enhancing interaction between cortisol secretion and sympathetic tone has been described *via* peripheral (Wurtman, 2002) and central mechanisms (Schulz et al., 2020). On the other hand, and as has been demonstrated during acute exposure to normobaric hypoxia in awake subjects (Mackenzie et al., 2008), taking a daytime nap of fewer than 90 min is accompanied by significant changes in EPO secretion. Unfortunately, our study design cannot answer if this response was due to hypoxia, sleep disturbances or both.

Unlike Philips et al. (2015) who found changes in cognitive performance with evaluations such as the Digit Span and Stroop tests after 30 min of exposure to hypoxia our results before the nap (60 min of hypoxia) did not reveal changes between normoxic and hypoxic conditions. Our results are more in agreement with Latshang et al. (2013) who also failed to evoke changes. Additionally, although it has been shown that after naps there is an enhancement of mental activity (Milner and Cote, 2009; Qian et al., 2020), our results only showed a slight improvement in cognitive performance. It is possible that, as has been proposed (McMorris et al., 2017), changes become apparent only when arterial oxygen delivery is below a certain threshold. Hence, although our volunteers had low oxygen saturation levels, other oxygen delivery determinants might have compensated for that. Nevertheless, the correlation between cognitive performance and hormone levels, particularly cortisol and EPO, stands out. We corroborated that tests including reaction times were correlated with the neuroendocrine response to hypoxic stress (Phillips et al., 2015; Pramsohler et al., 2019).

Our study has a comprehensive approach to daytime sleep and hypoxic stress phenomena, including simultaneous neural and endocrine biomarker measurements. We evaluated activity and sleep both within the laboratory and on the days surrounding the experiment. However, as a pilot study several constraints need to be recognized to be improved in more extended assessments. For example, although our sample size exceeds the minimum recognized for a pilot study (Julious, 2005), larger groups under each condition may render stronger conclusions. Nevertheless, as a cross-over study, a smaller sample size has similar power and effect size than a parallel-group study with a larger one (Berry et al., 2006). Also, and linked to the constraints of the sample size is the scope of our conclusions, given that volunteers were not randomly selected and rather recruited from the local community. For this reason, the sample was cloistered to a relatively young age group of lowlanders with similar ethnic characteristics. Extrapolation to other age groups, highlanders or ethnically distant inhabitants does not apply. As a more functional aspect, there are differences between daytime and nighttime sleep (see above), hence, the interpretation of our data is limited to patterns observed during the day. Future research could address these differences by extending the study sites and populations analyzed as well as the times of day when the experiments are conducted. Moreover, the fact that the volunteers had a sleep deprivation the night before the hypoxic experiment could have an additive effect (Tobaldini et al., 2014). However, previous studies documented the preservation of cortisol release and cognitive variables, even with more extended restrictions than ours, as long as it is acute (Voderholzer et al., 2012; Schaedler et al., 2018). It is also true that in real scenarios proposed in our altitude simulations, this association between hypoxia and sleep restriction is not uncommon. However, a more detailed dissection of this relationship could be examined in future works. Adding to the clustered sample, the specific and controlled conditions of the laboratory decrease the scope of our inferences. Hence, extrapolation to natural altitudes is somewhat limited, and a differential response in hypobaric hypoxia is still debated (Millet et al., 2012; Mounier and Brugniaux, 2012). Additionally, it is well known that humidity and temperature could be different from those presented here, and both factors have important effects on sleep (Manzar et al., 2012; Okamoto-Mizuno and Mizuno, 2012) and hypoxic stress (Mugele et al., 2021). On the other hand, although most of the measurements were made during late summer and autumn, seasonal modifications (light-dark ratio, barometric pressure, humidity, and temperature) could affect sleep and the neuroendocrine responses to stress (Suzuki et al., 2019). Because we analyzed an acute exposure, long-term compensatory mechanisms to hypoxic stress are out of the scope of our conclusions. Finally, although infradian mechanisms could partly explain the respiratory response of our volunteers contrasting to previous nocturnal sleep studies (Lombardi et al., 2013; Caravita et al., 2015), given the small sample size, it was not possible to categorize responses concerning the menstrual cycle phase.

## 5 Perspectives and Significance

This study demonstrates that taking a daytime nap, even in mild hypoxia and during a short time, triggers a classic neuroendocrine response to hypoxic stress in lowlanders. Those changes are accompanied by modifications in sleep architecture, cardiac autonomic modulation, and respiratory pattern with marked differences between men and women, ratifying the importance of considering sex as a central determinant in all adaptive physiological responses. However, such changes do not impact cognitive performance or sleep efficiency.

Given the above, we hope that this research will lead us to corroborate these findings under natural conditions. In that case, the benefits of oxygen supplementation during napping at altitude should be evaluated. Finally, the elucidation of the specific effects that ethnicity, and sex hormones and their oscillations have on daytime napping should be analyzed in detail.

## Data Availability

The data set of this study is available upon reasonable request by accredited researchers or institutions with a methodologically sound proposal.
